# Beneath the surface: When a locally aggressive squamous cell carcinoma is revealed to be a squamoid eccrine ductal carcinoma

**DOI:** 10.1016/j.jdcr.2025.08.010

**Published:** 2025-08-20

**Authors:** Chase Andrew Pitchford, Daniel Hyman, Joshua Brady, Amelia Momtazzadeh, Christina Henson, Jeffrey D. McBride, Lindsey Collins

**Affiliations:** a Mark Allen Everett Department of Dermatology, OUHSC, Oklahoma City, Oklahoma; b OU Health Stephenson Cancer Center — Radiation Therapy, Oklahoma City, Oklahoma

**Keywords:** adnexal carcinoma, eccrine carcinoma, squamous cell carcinoma, squamoid eccrine ductal carcinoma

## Introduction

Squamoid eccrine ductal carcinoma (SEDC) is a rare adnexal neoplasm that exhibits squamous differentiation with cellular atypia, keratinous cyst formation, and squamous eddies superficially, and eccrine ductal differentiation extending into desmoplastic stroma.^1^, ^2^, ^3^, ^4^ In 1997, the first 3 cases were described as elderly patients with nonspecific nodules on the head, neck, and extremities which showed both squamous and ductal features on histopathology.^5^ The average age of those diagnosed with SEDC is 75.7 years, with a male predominance.^3^ Although there are no definitive histologic criteria for diagnosing SEDC, the differential diagnosis should include squamous cell carcinoma (SCC), microcystic adnexal carcinoma, SCC with eccrine differentiation, porocarcinoma, and metastatic adenocarcinoma. Diagnosis is based on a combination of the tumor’s aggressive clinical features and supportive—though not pathognomonic—histologic features. Histopathologic examination is required to distinguish the superficial portion of SEDC from its common imitator, SCC, which is relatively less aggressive.^1^^,^^4^^,^^5^ Nearly half of reported SEDC cases are initially misdiagnosed which could be attributed to only superficial portions sampled via the common shave biopsy technique used to diagnose neoplasms of uncertain behavior of skin.^3^^,^^6^ In this article, we highlight a case where even with deeper excisional specimens, multiple histopathology evaluations were needed to obtain the correct diagnosis of SEDC.

## Case report

**Fig 1:**
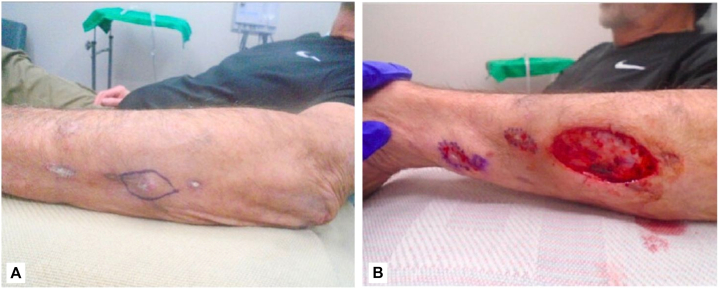
Wide local excision (WLE) of then diagnosed squamous cell carcinoma (SCC) with deep margin **(A)**. Subsequent Mohs defect and 2 marked ED&C sites and 1 marked punch biopsy site **(B)**. At this Mohs surgery visit and a subsequent visit, punch biopsies were performed of new lesions on the left arm. Pathology confirming invasive and well-differentiated SCC for both lesions. Excisions were performed, revealing squamoid eccrine ductal carcinoma with perineural invasion (Fig 2).

We present an 83-year-old white male formerly diagnosed with nonmelanoma and melanoma skin cancer who was referred to a dermatologic surgeon with biopsy-proven scattered invasive SCCs on the left forearm. The patient noted the appearance of these lesions 8 months prior but he had recently noticed an increase in size and number. Similar lesions were absent throughout the rest of his body. The recommended treatment was a wide local excision (WLE). The WLE pathology results indicated well-differentiated SCC with involvement of the deep margins. The patient elected to return for Mohs micrographic surgery. Upon evaluation at preoperation, a 5.0 cm linear scar on the left forearm was noted (Fig 1, *A*). The tumor was clear on the first Mohs stage (Fig 1, *B*). In the same visit, electrodesiccation and curettage were performed on 2 lesions based on a biopsy from the outside provider (Fig 1, *B*).

**Fig 2:**
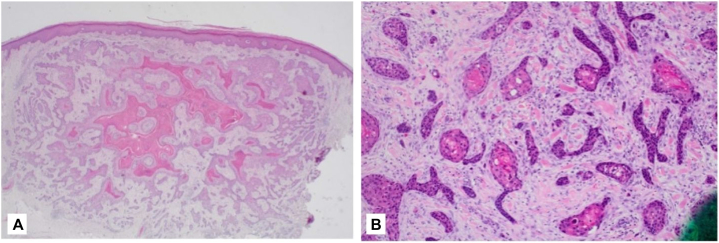
Excision specimen showing squamous cell carcinoma (SCC) **(A)**. Ductal differentiation was noted deep within the excision specimen **(B)**.

**Fig 3:**
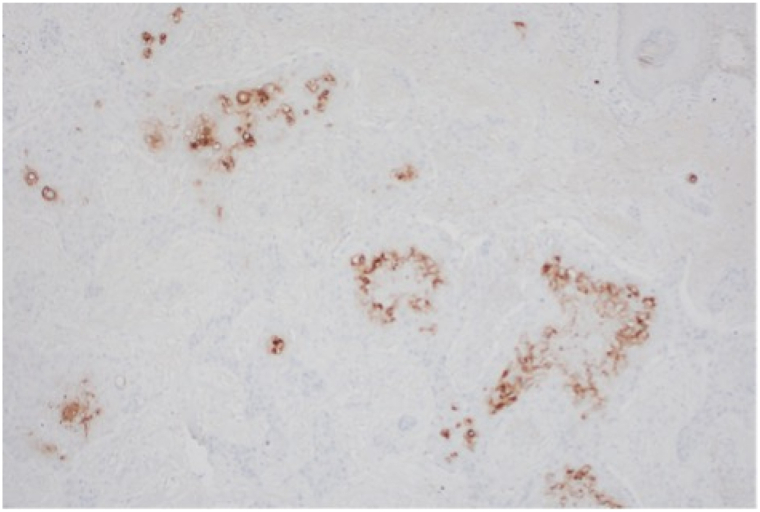
Ductal structures are highlighted by carcinoembryonic antigen (CEA).

One lesion showed positivity at the inferior margin and the other 0.2 cm from the deep margin. Immunohistochemistry demonstrated highlighted neoplastic cells with the P40 stain. Carcinoembryonic antigen and epithelial membrane antigen stains were positive throughout the ductal structures (Fig 3). To the authors’ knowledge, there is no single immunohistochemical stain that definitively distinguishes SEDC from SCC although certain histologic patterns can aid in diagnosis. In our case, carcinoembryonic antigen was more readily detectable in the eccrine tumor compared to what is typically seen in SCC. The presence of this stain, along with increased eccrine or luminal differentiation, absence of an epidermal connection, and a more aggressive growth pattern, supported the diagnosis.

The superficial portion of the biopsy specimen showed a squamoid derived carcinoma, but the deeper areas of the specimen showed diffuse ductal proliferation with perineural invasion. SEDC tumors often do not have an epidermal component due to their characteristic depth of spread. The morphologic characteristics in conjunction with the typical histologic nature of deep perineural invasion and glandular structures classically belonging to deep papillary and reticular dermis.

**Fig 4:**
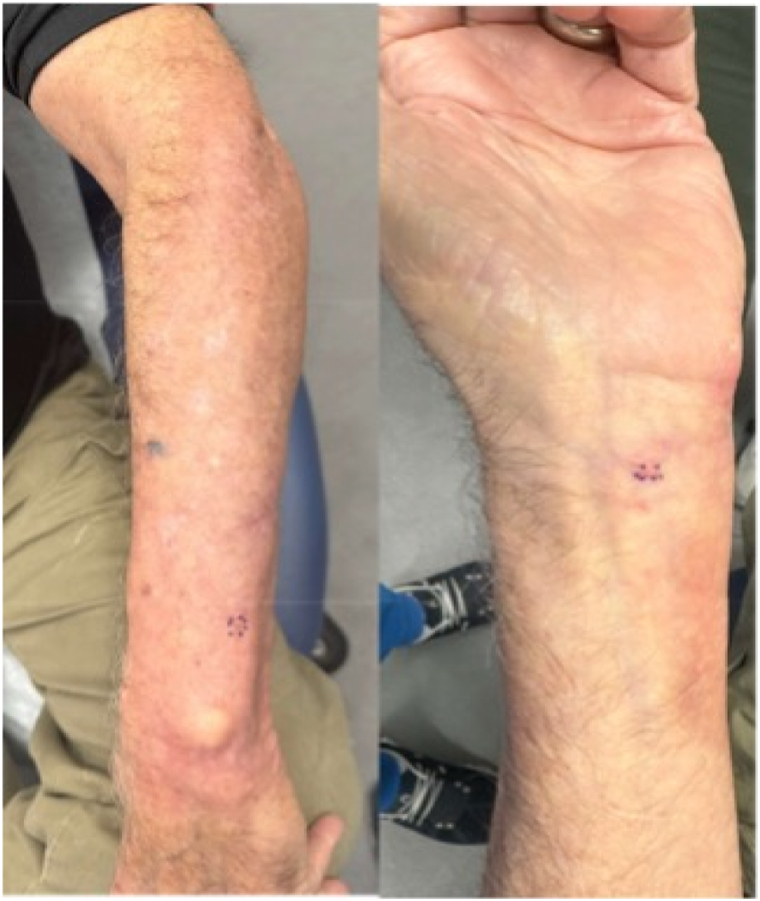
Four months following completion of radiation therapy, new dermal papules emerged, consistent with local recurrence and progression of the disease.

Ber-E4 was negative in neoplastic cells. The patient continued developing numerous dermal nodules throughout the left forearm ranging from 0.3 to 0.5 cm (Fig 4).

He was referred for radiotherapy due to inadequate clearance with surgery and innumerable new lesions. The patient completed an initial 4600 centigray over 20 fractions and an additional 1500 centigray over 6 fractions. Despite this, the patient had a recurrence both inside and outside the radiation field. An adipofascial turnover flap with split-thickness skin graft was performed by orthopedic surgery showing positive margins in all quadrants. A Caris test was performed to analyze relevant cancer biomarkers. The patient was found to have pathologic variants of biomarkers CDKN2A, TERT promoter, and TP53 were found. His tumor mutational burden was 48 suggesting high genomic instability and possible therapeutic benefit from pembrolizumab. Given the patient’s Caris test noting tumor mutational number of 48 in conjunction with FDA-approved pembrolizumab for tumors with tumor mutational number-high (>/ = 10mutations/megabase) solid tumors, pembrolizumab was considered as a viable treatment. At this time adjuvant radiation versus checkpoint inhibitor therapy is currently being discussed with the patient after his Caris test results.

## Discussion

Our case has described a locally aggressive squamous eccrine ductal carcinoma that was initially diagnosed as SCC. Given the malignant nature of SEDC and the presence of perineural invasion in our case, we propose that the tumor arose de novo and was not reactive to the prior procedures performed on the arm. Alternatively, the SEDCs, initially treated as SCC, may have exhibited unrecognized subclinical spread, consistent with the aggressive behavior, characteristic of the tumor. Moreover, the authors believe that there was 1 primary lesion and given SEDC’s tendency for metastasis, subclinical spread likely occurred hence the tumors complex disease course and necessity of multidisciplinary approach to care.

Etiologies for SEDC aside from SCC include eccrine carcinoma subtypes and biphasic carcinomas which are also under debate.^7^ SEDC is important to differentiate from other cutaneous tumors owing to its higher rate for morbidity including recurrence and metastases, likely due to its depth and predilection for perineural and lymphovascular invasion.^3^^,^^5^^,^^7^ Treatment options are similar to other forms of non-melanoma skin cancer. Following an average of 30.9 months of follow-up, Mohs micrographic surgery resulted in a lower local recurrence rate (0% to 5% of cases) than conventional excision (10% to 70% of cases) for SEDC.^4^^,^^8^ Thus, Mohs micrographic surgery should be considered for an SEDC treatment plan.

## Conflicts of interest

None disclosed.
